# An unusual case of Cowden syndrome associated with ganglioneuromatous polyposis

**DOI:** 10.1186/s13053-016-0051-8

**Published:** 2016-05-10

**Authors:** Steffen Pistorius, Barbara Klink, Jessica Pablik, Andreas Rump, Daniela Aust, Marlene Garzarolli, Evelin Schröck, Hans K. Schackert

**Affiliations:** Department of Visceral, Thoracic and Vascular Surgery, Technische Universität Dresden, Fetscherstr. 74, Dresden, 01307 Germany; Institute for Clinical Genetics, Technische Universität Dresden, Fetscherstr. 74, Dresden, 01307 Germany; Institute of Pathology, Technische Universität Dresden, Fetscherstr. 74, Dresden, 01307 Germany; Department of Dermatology, Technische Universität Dresden, Fetscherstr. 74, Dresden, 01307 Germany; Department of Surgical Research, Technische Universität Dresden, Fetscherstr. 74, Dresden, 01307 Germany; University Cancer Center (UCC) and Outpatient Clinic for Hereditary Gastrointestinal Tumors, Technische Universität Dresden, Fetscherstr. 74, Dresden, 01307 Germany; German Cancer Consortium (DKTK), Dresden, Germany; German Cancer Research Center (DKFZ), Heidelberg, Germany; National Center for Tumor Diseases (NCT), Dresden, Germany

**Keywords:** Ganglioneuromatous polyposis, Colon cancer, Cowden syndrome, *PTEN* germline mutation

## Abstract

**Background:**

Ganglioneuromatous polyposis (GP) is a very rare disorder which may be associated with other clinical manifestations and syndromes, such as Cowden syndrome, multiple endocrine neoplasia (MEN) type II and neurofibromatosis (NF) 1. The risk for malignant transformation of ganglioneuromas is unknown, and the combination of GP with colon cancer has been only very seldom reported.

**Methods and results:**

We report the case of a 60-year old male patient with adenocarcinoma, adenomas and lipomas of the colon and multiple gastroduodenal lesions combined with generalised lipomatosis and macrocephaly. Based on the initial endoscopic and histological findings, a (restorative) proctocolectomy was recommended but declined by the patient. Instead, a colectomy was performed. The histological examination revealed an unforeseen GP in addition to the colon cancer. Extensive molecular diagnostics allowed for the differential diagnosis of the causes of the clinical manifestations, and the clinical suspicion of Cowden syndrome could not be confirmed using Sanger Sequencing and MLPA for the analysis of *PTEN*. Finally, a pathogenic germline mutation in *PTEN* (heterozygous stop mutation in exon 2: NM_000314 (PTEN):c.138C > A; p.Tyr46*) could be detected by next-generation sequencing (NGS), confirming an unusual presentation of Cowden syndrome with GP.

**Conclusions:**

Cowden syndrome should be considered in cases of GP with extracolonic manifestation and verified by combined clinical and molecular diagnostics. Because GP may represent a premalignant condition, a surgical-oncological prophylactic procedure should be considered. Based on our experience, we recommend early implementation of Panel NGS rather than classical Sanger sequencing for genetic diagnostics, especially if various diagnoses are considered.

## Background

Ganglioneuromatous polyposis (GP) is a very rare disorder, and it may be associated with various clinical manifestations and syndromes such as Cowden syndrome, MEN II and NF1 [1–4]. The risk for malignant transformation of ganglioneuromas is unknown, and the combination of GP with colon cancer has been only seldom reported.

We report the case of a patient with GP, adenocarcinoma, adenomas and lipomas of the colon, multiple gastroduodenal lesions as well as generalised lipomatosis and macrocephaly, which was investigated through extensive clinical and molecular diagnostics, reporting the findings and reviewing the current literature.

## Case report

### Personal and family history

A 60-years old male caucasian patient was referred to the Outpatient Clinic for Hereditary Gastrointestinal Tumors at the University Cancer Center (UCC) Dresden after being diagnosed with polyposis coli. There was no personal history of malignancies, yet at the age of 33 a thyroid resection because of struma nodosa with an incidental finding of two microfollicular adenomas was performed. Retrospectively, he acknowledged having observed lipomas of the arms and torso since the age of 20. A significant comorbidity with hypertension, ischemic heart disease (myocardial infarction and coronary stents one year previously), tachyarrhythmia and hyperlipoproteinemia was reported.

**Fig. 1:**
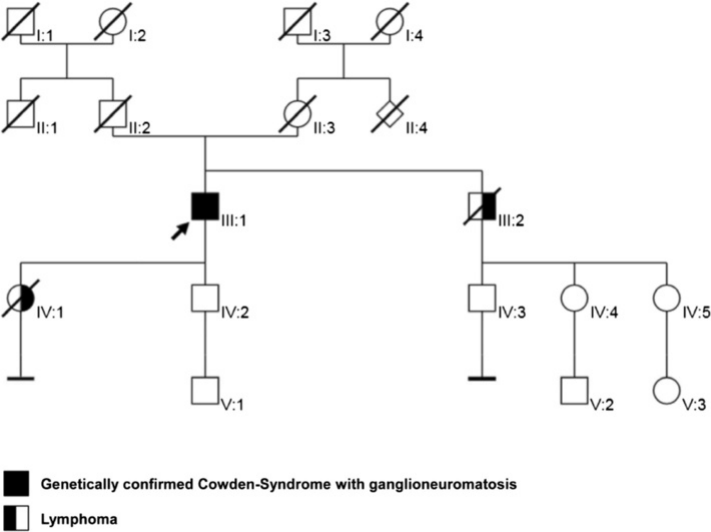
Pedigree. The index patient is indicated by the black arrow

His only brother died at age 48 due to an anaplastic T-cell lymphoma and his daughter died due to a lymphoma at age 30 (Fig. 1). No further family members including his parents were reported as suffering from malignant diseases.

### Preoperative findings

#### Physical/dermatological examination

**Fig. 2:**
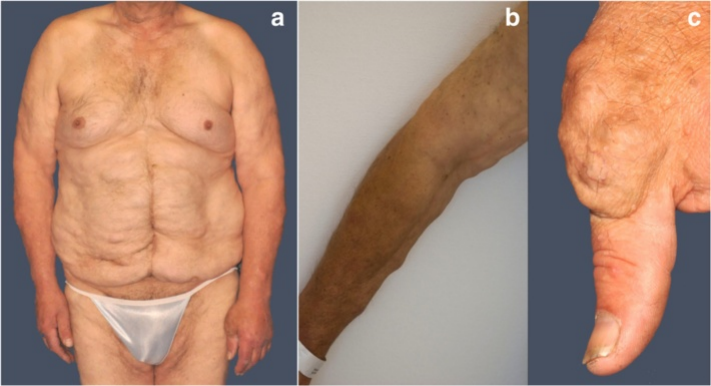
Multiple lipomas of various sizes on the torso (**a**), limbs (**b**) and on the left thumb basal joint right (**c**)

**Fig. 3:**
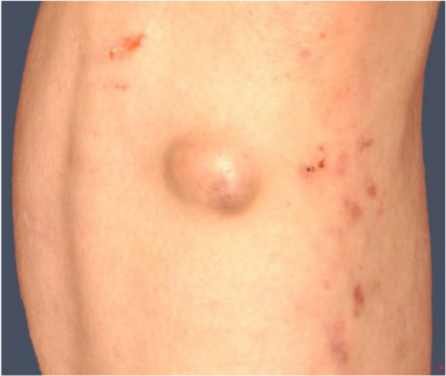
Solid, subcutaneous tumor (2.5 x 2.0 cm in size) at the lower leg

The physical examination revealed multiple soft, painless, subcutaneous nodules on the torso and limbs, which had progressively developed over the past decades. There were multiple lipomas of various size (the largest measuring approximately 6 x 4 cm), on the torso, limbs and on the basal joint of the left thumb (Fig. 2a–c). In addition, a solid, subcutaneous tumor (2.5 x 2.0 cm in size) was palpable at the lower leg (Fig. 3). Acral keratosis or facial papules such as trichilemmomas as well as papillomatous papules – typical manifestations of patients with a Cowden syndrome - were not observed. Apart from a mild lingua plicate and penile lentigiosis with irregular, pigmented macules affecting the penile glans, no other mucosal abnormalities, particularly no papillomatosis of oral mucosa were seen. There was a mild gynecomastia. Furthermore, the examination of the skin revealed sporadic verrucae seborrhoicae, melanocytic nevi and sporadic red-colored, flat papules on the torso in terms of senile hemangiomae. In addition, a macrocephaly (occipital frontal circumference (OFC) 64 cm (5 cm > 97.P) was measured.

#### Endoscopy

**Fig. 4:**
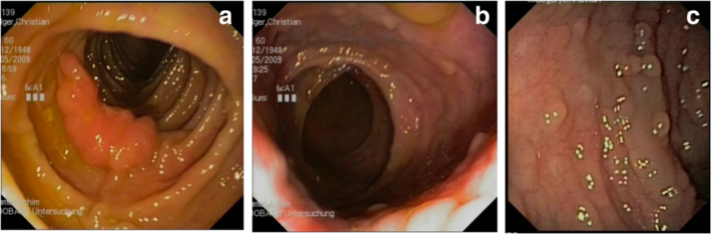
Preoperative colonoscopic views: tumor of the descending colon (**a**), polyps of sigmoid colon (**b**) and rectum (**c**)

A complete colonoscopy performed by the referring physician revealed a macroscopically malignant tumor of the descending colon near the splenic flexure, which in the histological examination showed a high-grade intraepithelial dysplasia as well as numerous polyps in the entire colon and rectum, later confirmed by biopsies as hyperplastic polyps and dysplastic adenomas (Fig. 4a–c).

**Fig. 5:**
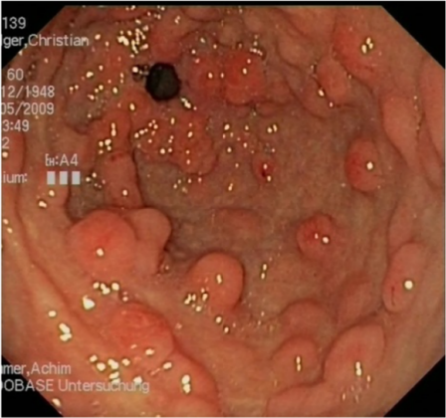
Preoperative gastroscopic view: gastric polypoid lesions

Furthermore, the upper-GI endoscopy revealed more than 60 gastric and about 25 duodenal polypoid lesions (Fig. 5). Histological examination of the gastric lesions had revealed distinctly regeneratory foveolar hyperplasia.

#### Radiological and nuclear imaging

The abdominal ultrasonography detected small ambiguous hepatic lesions which were suggestive of liver metastases and haemangiomas, which were also observed in the abdominal CT- and MRI-scans. No pathologic findings in other organs were seen. F-FDG PET-CT scan revealed a focal accumulation of radio-nucleotide in the left-sided upper abdomen, corresponding to the colonic tumor of the splenic flexure, and a parajugular enhancement corresponding to the left lobe of the thyroid gland; no evidence of any metastases of the colonic cancer was found (images not shown).

### Surgical treatment

With the work-diagnosis of an attenuated adenomatous polyposis coli or similarly atypical polyposis syndrome with a colon carcinoma in a 60-year old male, we recommended a (restorative) proctocolectomy, which was declined by the patient. Consequently, we opted for a total colectomy and lymphadenectomy (according to oncologic guidelines) with terminal ileostomy (Hartmann`s procedure) as the first step of the treatment. The final decision concerning further procedures shall be made depending on the postoperative tumor staging of the colon carcinoma and the clinical course of the disease. The patient tolerated the procedure rather well, and the suspicion of liver metastases could not be confirmed intraoperatively.

### Histopathology and immunohistochemical examination

Gross examination of the colectomy specimen revealed a 91-cm segment of colon with 3 cm of terminal ileum, the ileocecal valve and the appendix included. Throughout the colon (on the mucosa), there were more than 50 rounded-to-sessile polyps ranging from 0.3 to 0.5 cm in diameter. Two polyps in the ascending colon measured 1.5 cm. In addition, there was a flat lesion measuring 2.3 x 2.2 cm located in the splenic flexure, which on cross section had a whitish appearance.

**Fig. 6:**
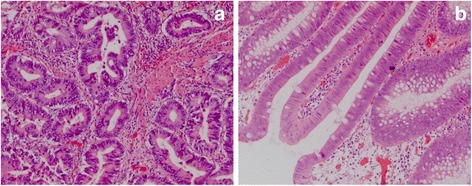
Adenocarcinoma of the descending colon (20x) (**a**) and tubular, low-grade dysplastic tubulo-villous colon adenoma with low grade dysplasia (20x) (**b**)

**Fig. 7:**
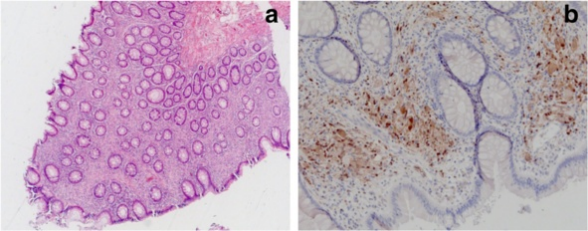
Ganglioneuroma in HE (4x) (**a**) and S 100 (20x) staining (**b**)

Histologic examination of the tumor revealed a moderately differentiated adenocarcinoma arising from a tubular adenoma, which infiltrated the lamina muscularis propria without invasion of the pericolonic adipose tissue (Fig. 6a). The examination of 26 mesocolic lymph nodes revealed no lymph node metastases, thus the stage defined for the tumor was pT2 N0 (0/26) M0 L0 V0 G2 R0. Histologic examination of the polyps revealed a large number of ganglions and spindle cells proliferating between glands, replacing the lamina propria. Immunohistochemical staining for S-100 protein was performed and showed strong reactivity in the spindle cell component, confirming the diagnosis of GP (Fig. 7). Additionally, there were two submucosal lipomas in the ascending colon and five tubular adenomas with low-grade dysplasia (Fig. 6b). The intestinal ganglioneuromas were confined to the mucosa. There were more than 50 sessile polypoid lesions, suggesting the diagnosis of ganglioneuromatous polyposis.

### Further diagnostics, clinical course and follow-up

As a result of the GP diagnosis, we attempted to establish a link between the clinical and histological manifestation and the phenotype of the patient with any of the known syndromes in which ganglioneuromas/ganglioneuromatosis/GP and other intestinal polyps may occur, such as Cowden syndrome, Multiple endocrine neoplasia type IIb, Neurofibromatosis von Recklinghausen type I, atypical familial adenomatous polyposis among others. Therefore, in addition to the preoperative staging, the following diagnostic procedures aiming at a differential diagnosis were performed:

- Measurement of parathormone, calcitonin, adrenalin and noradrenalin, which revealed no abnormal values;
- Ultrasonography of the thyroid gland and parathyroid glands, revealing a left-sided thyroid nodule but no enlarged parathyroid glands, which appeared as cold nodules in the scintigraphy. To rule out a malignancy, a fine-needle biopsy of the thyroid nodule was performed and was negative.
- MRI-scan of the brain revealing no intracranial tumor, yet left-sided hemangioma of the frontoparietal skull.

An overview of the clinical differential diagnostics is shown in Table 1.

**Table 1:** Clinical differential diagnosis

Syndrome	Pros	Cons	Conclusion
Cowden syndrome	2 major criteria: macrocephaly, multiple gastrointestinal ganglioneuromas; 2 minor criteria: thyroid gland lesion (adenoma, multinodular goiter), lipomas	no pathognomonic criteria (Cowden-typical mucocutaneous lesions, Lhermitte-Duclos disease)	diagnostic criteria fulfilled
Neurofibromatosis type I	ganglioneuromas	no neurofibromas, no café-au-lait spots, no gliomas	unlikely
Multiple endocrine neoplasia (MEN) type IIb	ganglioneuromas	no medullary thyroid cancer no pheochromocytoma no marfanoid habitus no neurofribromas	unlikely
Atypical/attenuated adenomatous polyposis coli (FAP) and other polyposis syndromes	colorectal adenomas and adenocarcinoma, gastric and duodenal polypoid lesions	multiple lipomas, ganglioneuromatous poylposis	unlikely

After extensive multidisciplinary postoperative counselling the patient decided to keep his residual rectum despite of the persistent multiple rectal ganglioneuromas, and therefore, an ileo-rectostomy was performed. We followed up the patient by sigmoidoscopy with rebiopsies of the persistent rectal ganglioneuromas to exclude malignant transformation, esophago-gastro-duodenoscopy and sonography every 6 and 12 months, respectively. After a follow up of 6 years, neither metastases, nor recurrence nor new primary malignancies were detected.

### Molecular analyses

After genetic counselling and obtaining informed written consent, molecular analyses were performed in order to further differentiate the patient`s phenotype and to identify a potential underlying germline mutation. Previous conventional genetic testing in 2010 revealed no germline mutations in known hereditary cancer syndrome genes that are associated with either polyposis or colon cancer. Given that the patient fulfilled the diagnostic criteria for Cowden Syndrome based on the National comprehensive Cancer Network (NCCN) 2010 criteria, and that he was above the threshold in the clinical scoring system proposed by Tan et al. [5], we performed firstly the investigation for germline mutations in the *PTEN* gene. All coding exons and the promotor region of *PTEN* were sequenced by Sanger-Sequencing of DNA extracted from peripheral blood lymphocytes, in which no pathogenic mutations were detected. Small deletions and duplications of *PTEN* were excluded using MLPA („Multiplex Ligation-dependent Probe Amplification“; Company Kit P225-B2). However, Sanger sequencing of DNA of paraffin-embedded tumor tissue of the colon carcinoma revealed a heterozygous somatic mutation in *PTEN*: NM_000314:c.388C > T; p.Arg130*, resulting in a stop-codon. No mutation in *PTEN* was found in the DNA of tissue of the ganglioneuroma. In addition, sequencing of all codons of additional genes potentially related to Cowden syndrome (*SDHB, SDHC, SDHD, Akt1* and *PIK3CA*) revealed no mutations. No pathogenic mutations could be found in the *RET*-protooncogene, causing MEN2 syndrome, and in *APC, MUTHY, BMPR1A* and *SMAD4* (sequencing and MLPA), responsible for different polyposis syndromes. To exclude HNPCC, testing for microsatellite instability and immunhistochemistry on tumor tissue was performed and revealed a microsatellite stable (MSS) colon carcinoma with normal expression of the mismatch repair proteins MLH1, MSH2, MSH6 and PMS2 (data not shown). The *NF1*-gene was not analyzed, because there were no clinical signs for neurofibromatosis Type 1 (no freckling, no café-au-lait-spots, no neurofibromas).

To exclude genome-wide small deletions and duplications, we performed a comparative genomic hybridization (CGH) array analysis using an Agilent 400 k microarray, which revealed no pathogenic deletions or duplications. Karyotype analysis on cultivated lymphocytes showed a normal male karyotype 46,XY.

**Fig. 8:**
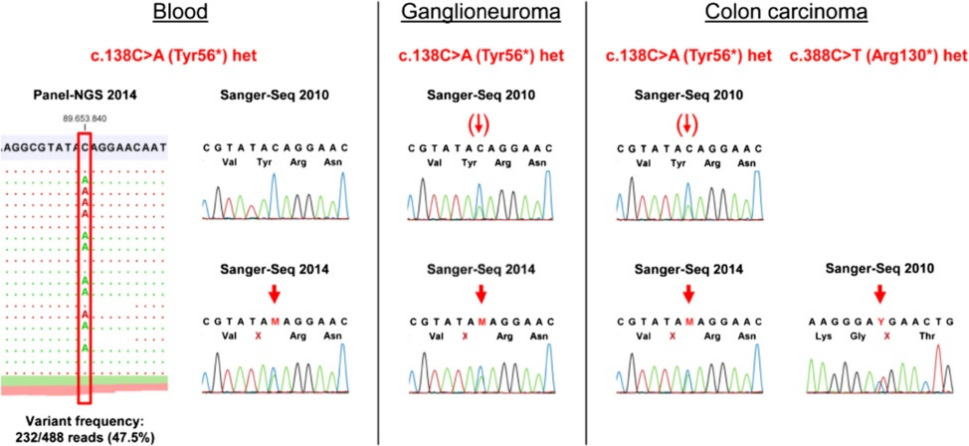
Molecular genetic analyses of *PTEN* on DNA from blood and tumor tissue from a ganglioneuroma and the colon carcinoma. Next-generation sequencing revealed a stop mutation in *PTEN*, NM_000314(PTEN):c.138C > A;p.Tyr56* in the patient’s blood in a heterozygous state indicated by the allele frequency of about 47.5 % (left). This mutation has not been seen in the first Sanger-Sequencing performed in 2010 (upper left), but was confirmed by a second Sanger-Sequencing in 2014 (lower left, indicated by arrow). The ganglioneuroma (middle) and the colon carcinoma (right) showed the mutation NM_000314:c.138C > A only as a small peak in the first Sanger-Sequencing, but in a heterozygous state in the second Sanger-Sequencing, thus indicating allele drop-out in the first Sequencing. The colon carcinoma additionally showed a second somatic mutation NM_000314(PTEN):c.388C > T;p.Arg130* indicating compound-heterozygous mutation of *PTEN* in the malignant tumor

Despite of the negative results of the previous molecular analyses in the germline, we assumed a Cowden syndrome based on the patient’s clinical signs and manifestations. Since a somatic pathogenic heterozygous mutation was found in the colon carcinoma, we speculate that this might be the second hit in *PTEN*, while the first hit, probably the constitutional mutation, could not be found with the previously used methods. Therefore, when next-generation sequencing technique became available, we repeated the analysis on DNA from patient’s blood using the Illumina TruSight Cancer Panel target enrichment protocol (Illumina Inc., San Diego, CA, USA) which covers 1737 exons of 94 cancer-relevant genes as recently described [6]. We identified a heterozygous nonsense mutation in exon 2 of *PTEN*, NM_000314:c.138C > A; p.Tyr46*. The Sanger-Sequencing for *PTEN* Exon 2 was repeated on DNA from patient’s blood, as well as from tissue of the ganglioneuroma and the colon carcinoma and this time the mutation NM_000314:c.138C > A was confirmed in all samples in a heterozygous state (Fig. 8). Re-evaluation of the firstly performed Sanger-sequencing data revealed a small second peak at position c.138 in DNA from the ganglioneuroma and the colon carcinoma, but not from the patient`s blood (Fig. 8).

## Discussion

### Molecular analyses

Considering the phenotype and the pathological findings, the most likely clinical diagnosis in our patient was Cowden syndrome. However, no pathogenic *PTEN*-mutation was detected in the germline at initial diagnosis in 2010 using Sanger-Sequencing and MLPA, only later when using next-generation sequencing and then confirmed using Sanger Sequencing. Based on our findings, allele drop-out is the most likely explanation for the false-negative result. Allele drop-out is a major concern of diagnostic sequencing using primer-based enrichment (such as used in Sanger-Sequencing), since one variant within a primer is sufficient to cause drop-out [7]. Using new target-enrichment methods and next-generation sequencing (NGS) strategies these false-negative results can be avoided [8]. Moreover, the step-wise analyses using Sanger-Sequencing of the multiple genes that account for differential diagnoses is time-consuming and expensive. All genes analyzed in our case by Sanger-Sequencing are also represented on the Illumina TruSightCancer Panel and hence can be simultaneously tested in a single analysis. In the future, this approach will allow for diagnosing more patients correctly, especially in patients with atypical phenotypes. Therefore, it cannot be excluded that other “unusual” cases in the literature without identified mutations in known genes must be considered as false-negatives. In our opinion, transition of Sanger-Sequencing to a target NGS based test would not only increase the reliability of diagnosis but would also cut the turn-around time and costs and improve genetic diagnosis especially in cases with unusual disease manifestations and/or in cases with multiple differential diagnoses.

### Clinical aspects

Shekitka et al. proposed to divide ganglioneuroma formations into three groups: polypoid ganglioneuromas, diffuse ganglioneuromatosis and GP [3]. GP is a very rare disorder and may be associated with different syndromes and other preconditions [1–4]. In addition, some patients with a GP also develop adenomatous, other hamartomatous or juvenile colon polyps [2, 9–15]. The combination of GP with adenocarcinomas of the colon has been reported in a few cases only [3, 12, 16–18]. Although single ganglioneuromas are not unusual in patients with Cowden syndrome [10, 19–21], GP in Cowden syndrome is a rarity [1, 10, 18, 19] and may result in delayed diagnosis in patients with Cowden syndrome [22].

Very similar to our case, a 41-year-old patient with GP, colon adenomas and cutaneous lipomas was reported by Chan et al. in 2006 [10], however genetic testing was not performed. In 2013, Vinitsky et al. reported a 25-year-old woman who as a teenager showed macrocephaly and multiple gastrointestinal lesions including ganglioneuromas, hamartomas, lipomas, juvenile, and hyperplastic polyps in association with extra-intestinal tumors including a retroperitoneal lipoma, storiform collagenoma, and a fibrolipomatous hamartoma. This patient had not developed a malignant tumor yet. *PTEN* mutation analysis identified a deletion in exon 2, confirming the diagnosis of Cowden syndrome [22]. In addition, in 2012 Trufant et al. presented the case of a 42-year-old man with colonic ganglioneuromatous polyps and an adjacent colonic adenoma giving rise to a signet-ring adenocarcinoma with lymph node metastases in the setting of Cowden syndrome due to a pathologic nonsense mutation at the *PTEN* locus. Unfortunately, the location of the mutation in *PTEN* was not given [18].

Accordingly, the pertinent question is whether there is a molecular basis for such an unusual phenotype of GP in patients with Cowden syndrome. Supposedly, a germline nonsense mutations (or mutations) in the first exons of *PTEN* could be associated with a severe phenotype. Marsh et al. found that in patients with Cowden syndrome, only 33 % of the mutations in *PTEN* were nonsense mutations, while mutations in the first three exons are rare [23]. Although genotype-phenotype analyses were performed in this study, no data were presented concerning the association with GP. However, deletion of *PTEN* has been shown to be associated with ganglioneuromatosis in the mouse [24]. Our patient as well as the two patients with genetically confirmed Cowden-Syndrome combined with ganglioneuromatosis in the literature carried either nonsense mutations or a partial deletion of *PTEN*.

Based on the mentioned phenotype and the reported cases of a combination of GP with adenocarcinomas of the colon [3, 12, 16–18], it is feasible that ganglioneuramatous polyposis is a pre-malignant condition, an opinion shared by other authors [12].

Finally, the key issue is which surveillance program and what kind of prophylactic procedure should be recommended to a patient with GP. Kanter et al. recommended a proctocolectomy because of the pre-malignant condition [12]. However, it is well-known that in those patients, particularly the elderly, the quality of life after this procedure (even if performed as a restorative proctocolectomy) is worse than in patients with a colectomy and ileo-rectostomy. In our patient, we initially recommended a (restorative) proctocolectomy, assuming that an attenuated adenomatous polyposis coli or another atypical polyposis syndrome with a colon carcinoma was present. However, the patient refused this procedure. Whether or not his decision will have consequences in the future clinical course remains unclear, but at least in the 6-year follow-up neither metastases, recurrence nor new primary malignancies were detected in the surveillance programm.

## Conclusions

If GP with extracolonic manifestations is diagnosed, Cowden syndrome should strongly be suspected and ascertained by combined clinical and molecular diagnostics. Based on our experience, we recommend the early implementation of Panel NGS rather than classical Sanger sequencing for the genetic diagnosis, especially if various differential diagnoses are assumed. Because GP could represent a pre-malignant condition, a prophylactic surgical oncologic procedure should always be considered.

## Consent

The patient`s diagnosis, treatment, councelling and surveillance was performed following the principles of medical ethics. An ethics approval with by the ethics committee was not required.

Written informed consent was obtained from the patient for publication of this case report and any accompanying images. A copy of the written consent is available for review to the Editor-in-Chief of this journal upon request.

## Abbreviations

F-FDG PET-CT: Positron emission tomography with 2-deoxy-2-[fluorine-18] fluoro-D-glucose integrated with computed tomography
GP: ganglioneuromatous polyposis
HNPCC: hereditary nonpolyposis colorectal cancer
MEN: multiple endocrine neoplasia
NF: neurofibromatosis
NGS: next generation sequencing
MLPA: multiplex ligation-dependent probe amplification
